# D2/D3 Receptor Agonism: Paving the Way for a New Therapeutic Target for Taste Disorders in Parkinson’s Disease and Other Conditions?

**DOI:** 10.1093/ijnp/pyac042

**Published:** 2022-07-31

**Authors:** Elisa Mantovani, Stefano Tamburin

**Affiliations:** Department of Neurosciences, Biomedicine and Movement Sciences, Neurology Section, University of Verona, Italy; Department of Neurosciences, Biomedicine and Movement Sciences, Neurology Section, University of Verona, Italy

**Keywords:** D2/D3 receptor agonism, Parkinson’s disease, pramipexole, taste disorders, treatment

## Abstract

Chemosensory (i.e., olfaction and taste) dysfunction is common in neurodegenerative (e.g., Parkinson’s disease, Alzheimer’s disease, and dementia), psychiatric (e.g., depression, bipolar disorders, other conditions), and postinfectious (i.e., long COVID) diseases and in the elderly. Despite its impact on patients’ quality of life, no established treatment for taste disorders exists so far. A recent report on the effect of pramipexole, a D2/D3 agonist, on taste performance in healthy participants provides support for a new potential therapeutic target for taste dysfunction to be tested in future randomized, placebo-controlled, clinical trials across several populations reporting gustatory symptoms.

Chemosensory (i.e., olfaction and taste) function is mediated by highly specialized sensory systems with peripheral and central subdivisions. The olfactory epithelium and the bundles of olfactory receptor axons project to the olfactory bulb, which is connected to the primary olfactory cortex, orbitofrontal and insular cortices, thalamus, and hippocampus (Smith and Bhatnagar, 2019). Peripheral taste receptor cells are widely distributed across the oral cavity, pharynx, larynx, and upper esophagus and are connected to the rostral division of the nucleus of the solitary tract in the medulla oblongata that projects to the thalamus, orbitofrontal, insular, and anterior cingulate cortices, amygdala, and hypothalamus (Vincis and Fontanini, 2019). The neuropharmacology of taste has been less extensively studied in humans because most studies dealt with the role of dopaminergic signaling in reward-related behaviors to gustatory stimuli (Baik 2013).

Olfaction and taste provide critical information about the environment and play a key role in appetite, food choices, and nutrient intake by detecting potentially appetitive or dangerous compounds, indicating food’s nutritional values, and influencing the quantity of food ingested throughout specific signals of satiety (Kershaw and Mattes, 2018). Chemosensory impairment can alter food choices and intake, lead to exacerbation of disease states, impair nutritional status and immunity, and result in unintended weight loss (Sergi et al., 2017).

Parkinson’s disease (PD) is a progressive neurodegenerative disorder whose hallmark is progressive loss of dopaminergic neurons, particularly those within nigrostriatal projections. The resultant dopamine deficiency within the basal ganglia circuitry, together with the involvement of other neuromodulators (i.e., serotonin, norepinephrine, acetylcholine, adenosine, gamma-aminobutyric acid, glutamate, and histamine) lead to several motor and non-motor symptoms, some of which may precede PD clinical diagnosis even by decades (Kalia and Lang, 2015).

Non-motor symptoms have a marked impact on a patient’s quality of life (QoL) and add to the overall burden of PD morbidity (Seppi et al., 2019). Non-motor symptoms are highly prevalent along the disease course, with more than 95% of patients being affected by at least 1 non-motor symptom since the initial PD stages, but their treatment remains highly challenging and often inadequate (Seppi et al., 2019).

Olfaction and taste dysfunction are non-motor features of PD that have been extensively documented (Doty and Hawkes, 2019). Reduced olfaction has been consistently reported since PD preclinical stages, when alpha-synuclein deposition, the neuropathological hallmark of PD, can be demonstrated in the olfactory mucosa (Stefani et al., 2021). Hypogeusia (i.e., diminished taste), despite being less studied and often milder than olfactory deficits, as most patients are unaware of this symptom, has been reported both in the prodromal and clinically established phases of PD. In the ONSET PD study, one of the largest longitudinal reports on preclinical PD, taste loss was reported 2–10 years before PD diagnosis (Pont-Sunyer et al., 2015). A prospective case-control study found that 28.6% and 3.8% out of 474 patients with combined olfactory and gustatory disorder and pure gustatory deficit, respectively, developed PD within 8.1 years after the first chemosensory assessment (Haehner et al., 2019). According to these reports, taste dysfunction has been suggested as an early, slowly progressive feature of PD along with olfactory dysfunction (Cecchini et al., 2015; Pont-Sunyer et al., 2015; Nigam et al., 2021). Brain regions involved in taste processing (e.g., nucleus of the solitary tract, operculum, insula, orbitofrontal cortex) are indeed affected by PD neuropathology (Cecchini et al., 2015). The neurochemistry of taste has been seldom explored in PD, but drugs acting on dopaminergic receptors may be potential key players for the treatment of taste dysfunction, a still unmet need (Doty 2019).

To probe the putative causal role of dopamine in taste processing, Kaltenboeck and colleagues (2022) performed an experimental medicine study on 40 healthy participants (age: 18–43 years) who underwent psychophysical taste assessment to explore taste recognition and pleasantness/disgustingness to appetitive/aversive taste samples 12–15 days after treatment with 1.0 mg/d pramipexole, a D2/D3 receptor agonist, or placebo. This sample was recruited to take part in a larger experimental medicine study to explore depression-relevant neurocognitive effects of subacute pramipexole treatment. The authors found (1) enhanced taste recognition performance (i.e., higher total recognition accuracy) in pramipexole-treated participants compared with the placebo group; and (2) an association between pramipexole treatment and blunted response to both appetitive and aversive taste samples, but this pattern was linked to the experience of nausea, a side effect of pramipexole. The results of this study may have several clinical implications.

According to the lines of reasoning reported above, symptomatic treatment of taste dysfunction may improve patients’ QoL in PD. Taste alterations in PD may contribute to decreased food intake and weight loss that may predate motor symptoms (Akbar et al., 2015) and is associated with malnutrition, bone fractures, and cognitive decline (Kim et al., 2012; Ma et al., 2018). Given the strict interrelation between taste alterations and these important clinical outcomes, finding potential new treatments for taste dysfunction in PD may have relevant clinical impact.

Chemosensory abnormalities, in particular olfactory alterations, have been reported to a varying extent in other neurodegenerative diseases and dementia (e.g., Alzheimer’s disease, frontotemporal degeneration, vascular dementia) in addition to PD (Sakai et al., 2017; Doty and Hawkes, 2019). Gustatory changes involve the prodromal stages (Schiffman et al., 2002; Pont-Sunyer et al., 2015; Haehner et al., 2019; Nigam et al., 2021) and predict disease progression (Cecchini et al., 2015; Contri-Degiovanni et al., 2020) and cognitive decline (Doty and Hawkes, 2019), making them potentially useful candidate clinical biomarkers. Understanding the neuropharmacology of taste dysfunction in neurodegeneration may improve our pathophysiological insight of these clinical conditions. The findings by Kaltenboeck and colleagues (2022) offer some interesting hints on a potential involvement of dopaminergic transmission in taste processing. Whether taste dysfunction is responsive to dopaminergic therapies across different neurodegenerative diseases and whether these drugs may improve other symptoms because of a shared pathophysiology and neuropharmacology are topics of future clinical research.

The sense of taste, along with olfaction, is a topic of growing research interest in the field of normal and pathological aging. Taste impairment has been reported as highly prevalent in older adults and to contribute to age-related anorexia (i.e., a decrease in appetite and food intake in the elderly), which in turn represents a modifiable risk factor for frailty, sarcopenia, and mortality (Tan et al., 2020). Gustatory dysfunction was reported to predict cognitive decline in older adults (Churnin et al., 2019). Whether treatment of taste loss in the elderly through D2/D3 receptor agonists may be associated with a reduction in the odds of frailty and cognitive decline warrants future attention.

Chemosensory alterations have been reported in some psychiatric disorders, such as unipolar and bipolar depression (Kazour et al., 2017). Taste enhancement by D2/D3 agonists may improve symptoms of depression, as shown by a recent systematic review and meta-analysis on pramipexole (Tundo et al., 2019). Olfactory symptoms in depression might be another pharmacological target for dopamine agonists. Despite a heterogeneity of findings, atypical processing of odor and taste stimuli was reported in several neurodevelopmental disorders, notably in autism spectrum disorders (Boudjarane et al., 2017). Some degree of chemosensory dysfunction, particularly in gustation, was documented in patients with eating disorders (anorexia and bulimia nervosa) (Leland et al., 2022). Hedonic response to taste was found to be altered in patients with functional movement disorders, with no correlation to anxiety, depression, or alexithymia scores (Cecchini et al., 2020).

Chemosensory changes are also common in people with long COVID, a post-acute neurological condition characterized by several symptoms (e.g., memory/cognitive disturbances, post-exertional malaise, fatigue, sleep disturbances, headache, loss of smell or taste) that are unresponsive to conventional therapies and show detrimental consequences on patient’s everyday life and QoL (Mantovani et al., 2021; Nolen et al., 2022). No established treatment, except a wait-and-see approach, is available in long COVID. Whether D2/D3 receptor agonists may be useful also for such condition is another topic that deserves particular attention.

In conclusion, the report by Kaltenboeck and colleagues may offer support to testing D2/D3 receptor agonism for the treatment of taste dysfunction across a wide variety of clinical conditions (i.e., PD, other neurodegenerative disorders, depression, other psychiatric and neurological conditions, long COVID) and in elderly people. Double-blind, placebo-controlled, randomized clinical trials are needed to test this hypothesis. Open questions include whether dopamine agonists with different receptor affinity profile may show differential effects on taste dysfunction and may be more useful as mono- or add-on therapies. Future randomized clinical trials should offer information on the optimal dosage to treat taste dysfunction, preventing at the same time the onset of drug-related side effects (e.g., nausea, impulse control disorders, and related behaviors).

## Interest Statement

Authors have no financial interests to disclose.
